# ﻿The native species of *Callianthe* (Malvaceae, Malvoideae) in northern South America (Colombia, Venezuela, the Guianas and adjacent Brazil)

**DOI:** 10.3897/phytokeys.260.154906

**Published:** 2025-07-23

**Authors:** Laurence J. Dorr

**Affiliations:** 1 Department of Botany, MRC-166, National Museum of Natural History, Smithsonian Institution, P.O. Box 37012, Washington, D.C. 20013–7012, USA National Museum of Natural History, Smithsonian Institution Washington United States of America

**Keywords:** *Abutilon*, *Callianthe*, Colombia, Guyana, Malvaceae, Malvoideae, Venezuela

## Abstract

The native species of *Callianthe* Donnell (Malvaceae, Malvoideae) in northern South America (Colombia, Venezuela, the Guianas and adjacent Brazil) are reviewed. Five species are recognised, of which one, *C.sylvatica* (Cav.) Dorr, is widespread and found not only in Colombia (Cordillera Orientale) and Venezuela (Cordillera de la Costa and Sierra Nevada de Mérida), but also Andean Ecuador, Peru and Bolivia. The other four species have more restricted geographic ranges: *C.clarkei* Dorr, **sp. nov**. is known only from the Wassarai [Wassari] Mountains of southern Guyana; *C.petiolaris* (Kunth) Donnell occurs only in the Cordillera Orientale of Colombia; and *C.roseangelae* Dorr, **sp. nov.** is restricted to the Sierra Nevada de Mérida of Venezuela. A dearth of wild collections hampers establishing the precise native range of *C.insignis* (Planch.) Dorr, **comb. nov.**, which was discovered in the Andes of either Colombia or Venezuela and quickly entered cultivation. A Brazilian species, *Sidaspeciosa* Willd. ex Spreng. (≡ *S.rosea* Link & Otto), erroneously purported to occur in Venezuela, belongs in *Callianthe* and is recognised here as a synonym of *C.purpurascens* (Link) Dorr, **comb. nov.** Nine typifications are designated for synonyms of *Callianthe* species; seven are for names in *Abutilon* Mill. and two for names in *Sida* L.

## ﻿Introduction

*Callianthe* Donnell (Malvaceae, Malvoideae) is a genus of 50 (or more) species found in Mexico, Central America and South America. Species diversity is highest in Brazil where there are 36 species (34 endemic) (Flora e Funga do Brasil 2025) and, within that country, diversity is concentrated in the Atlantic Forest (Donnell et al. 2012; Takeuchi et al. 2014). While identifying material for a treatment of the Malvaceae for the Flora of the Guianas (see also Dorr (2022)), a new species, *C.clarkei* Dorr, sp. nov., known only from the Wassarai [Wassari] Mountains of southern Guyana, was recognised. Expanding a review of the genus to neighbouring countries, an additional four species of *Callianthe* were recognised as native to northern South America (Colombia, Venezuela, the Guianas and adjacent Brazil). *Callianthesylvatica* (Cav.) Dorr is widespread and found not only in Colombia (Cordillera Orientale) and Venezuela (Cordillera de la Costa and Sierra Nevada de Mérida), but also Andean Ecuador, Peru and Bolivia. The other native species found in northern South America have more restricted geographic ranges; *C.petiolaris* (Kunth) Donnell occurs only in the Cordillera Orientale of Colombia; *C.roseangelae* Dorr, sp. nov. is restricted to the Sierra Nevada de Mérida of Venezuela; and *C.insignis* (Planch.) Dorr, comb. nov. is found in the Andes of either Colombia or Venezuela. The precise distribution of this last species is unknown although, after it was discovered, it quickly entered cultivation. Flora e Funga do Brasil (2025) does not report *Abutilon* Mill., *Bakeridesia* Hochr. or *Callianthe* from Amapá, Amazonas, Pará or Roraima, the four Brazilian states that border the area of our concern.

The genus *Callianthe* was erected by Donnell et al. (2012) to accommodate most of the pluriovulate species of *Abutilon* and Bakeridesiasubg.Dipteron Hochr., which formed a clade based on ITS data (including the synapomorphy of a 25-base pair deletion in ITS2). Donnell et al. (2012) argued that *Callianthe* differed from *Abutilon* s.str. and *Bakeridesia* s.str. in having the following combination of morphological characters: 4–13 ovules per carpel, petals with deeply impressed veins, a glabrous staminal column, and a sparsely stellate-pubescent inner wall of the mericarp. Ovule number, however, is not a mutually exclusive character as several species of *Abutilon* have as many as six and *Bakeridesia* has as many as seven ovules per carpel. Nor is vestiture a mutually exclusive character. Both *Abutilon* and *Bakeridesia* have glabrous staminal columns and, in both genera, the inner wall of mericarps is glabrous (Donnell et al. 2012: table 1). The description of a new species of *Callianthe* from Venezuela (see below) indicates that the staminal column in this genus can be pubescent and, while the inner carpel walls of most species of *Callianthe* are pubescent, the new species from Venezuela has glabrous carpel walls. The only unique morphological character cited by Donnell et al. (2012) to define *Callianthe* thus appears to be the impressed petal veins that often are a different colour from the petals themselves. Although difficult to articulate in words, not only are the petal veins of *Abutilon* s.str. and *Callianthe* different, but so too is the overall aspect of the flowers. The petals of the former are more or less rotate, while those of the latter are strongly imbricate at anthesis and, although united only at the base the corolla, appear globose. In those species of *Callianthe* where the petal apices are spreading, the bases remain strongly imbricate.

The genus *Callianthe* includes the “flowering maples” or “Abutilons” of the horticultural trade. *Abutilon* s.str. is seldom cultivated commercially. In addition to the native species of *Callianthe*, several taxa (species, hybrids and cultivars) are grown in northern South America, especially in Colombia and Venezuela. These taxa occasionally escape or otherwise appear native. They will be discussed in a separate paper.

## ﻿Results

### ﻿Key to the native species of *Callianthe* in northern South America (Colombia, Venezuela, the Guianas and adjacent Brazil)

**Table:** 

1	Petioles longer than the leaf blades; leaf blade margin coarsely dentate; stems, petioles and peduncles with long simple hairs	***C.petiolaris***
–	Petioles shorter than the leaf blades; leaf blade margin crenulate or denticulate to subentire; stems, petioles and peduncles stellate-pubescent, lacking long simple hairs	**2**
2	Scandent shrubs; flowers pendulous, borne on long slender peduncles; petals pink or whitish with prominent and contrasting purplish-red or deep rose branching veins (petals drying dark red)	**3**
–	Erect shrubs; flowers ± erect, drooping or pendent on relatively stout peduncles; petals pale yellow with prominent veins (petals drying pink or orange and then veins more visibly contrasting)	**4**
3	Apical and non-apical leaf blades entire or very faintly 3-lobed, ± the same shape; calyx tubular or tubular-campanulate; staminal column glabrous	***C.insignis***
–	Apical leaf blades entire, non-apical leaf blades 3-lobed with apices of lateral lobes almost recurved; calyx campanulate or broadly-campanulate; staminal column stellate-pubescent	***C.roseangelae***
4	Leaf blades ovate or ovate-oblong, apices acuminate; flowers 1(–2) per axil; peduncles ± equal to petioles in length; petals 2–2.5 cm long	***C.sylvatica***
–	Leaf blades broadly ovate, apices acute to broadly acute, flowers (2–)3 per axil; peduncles exceeding petioles in length; petals ca. 1.2 cm long	***C.clarkei***

### ﻿Taxonomy

#### ﻿1. Callianthe clarkei

Taxon classificationPlantaeMalvalesMalvaceae

Dorr sp. nov.

19AD11B1-AEFD-5513-BE58-03A303836658

urn:lsid:ipni.org:names:77366028-1

Fig. 1

##### Diagnosis.

Differs from *Callianthesylvatica* (Cav.) Dorr in having broadly ovate (versus ovate or ovate-oblong) leaf blades with acute or broadly acute (versus acuminate) apices, axillary flowers (2–)3 per axil (versus solitary or paired), peduncles ± equal to (versus exceeding) petioles in length and petals 1.2 (versus 2–2.5) cm long.

**Figure 1.**
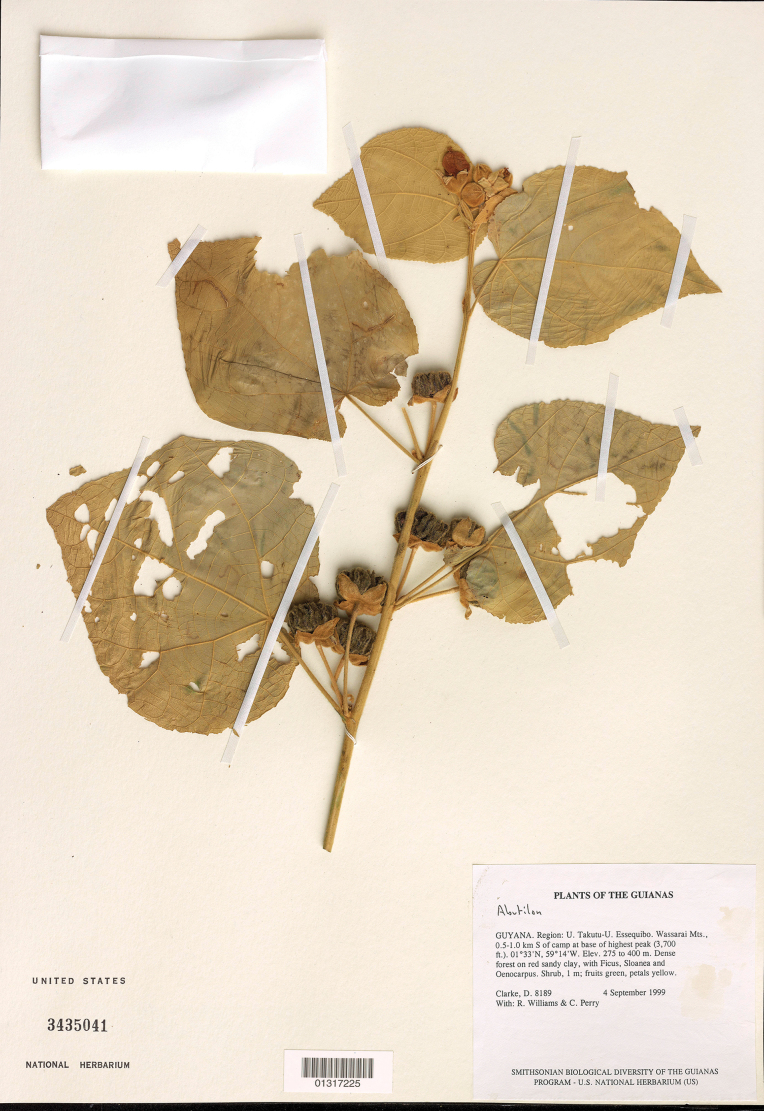
Holotype specimen of *Calliantheclarkei* Dorr (*Clarke et al. 8189*, US [01317225]).

##### Type.

Guyana • Upper Takutu-Upper Essequibo Region: Wassarai [Wassari] Mts., 0.5–1 km S of camp at base of highest peak, 01°33'N, 059°14'W, 275–400 m elev., 4 Sep 1999 (fl, fr), *H.D. Clarke, R. Williams, & C. Perry 8189* (holotype: US [01317225]!; isotypes: BRG-n.v., GENT-n.v., TEX-n.v.).

##### Description.

Erect shrubs, to 1 m tall; stems densely stellate-pubescent. Leaves simple, entire, leaf blades broadly ovate, very faintly 3-lobed, slightly asymmetrical in shape, decreasing in size towards the stem apex, 7–11 × 5–11 cm, bases cordate with a wide sinus, margin denticulate to subentire, teeth rounded (not sharp), apices acute to broadly acute, palmately 5–7-nerved from the base, mid-rib and 2° veins prominent below, slightly raised above, densely stellate-pubescent below, heterotrichous with a bed of smaller stellate hairs overlain by larger stellate hairs, lamina not visible, sparsely stellate-pubescent to glabrate above, lamina visible; petioles 2.5–4 cm long, densely stellate-pubescent; stipules falcate, ca. 10 × 1 mm, densely pubescent, caducous. Inflorescences axillary, (2–)3-flowered; peduncles 2–4.5 cm long, ± equal to or shorter than petioles in length, articulated ca. 2–3 mm below calyx. Floral buds globose, densely yellowish villous, sepals valvate. Involucel absent. Calyx campanulate, gamosepalous, 5-lobed, ca. ½ or slightly more divided at anthesis, lobes broadly triangular, 1 × 0.6 cm, densely stellate-pubescent externally, stellate-pubescent internally near margin, nectariferous at base. Petals rotund, ca. 1.2 × 1.2 cm, claw short, ca. 2 mm long, yellow, external surface with minute, whitish, multicellular hairs, inner surface glabrous. Staminal column ca. 1.2 cm long, glabrous; filaments clustered apically, ca. 3 mm long. Styles slightly exceeding the anther mass in length. Stigmas capitate. Schizocarps exceeding the calyx, 1.5 × 2 cm, ± oblate, stellate-pubescent. Mericarps ca. 8, unilocular, 4-seeded, rhomboid, ca. 1.2 × 0.8 cm, outer wall densely pubescent, inner wall sparsely stellate-pubescent, especially towards margin. Seeds teardrop-shaped, ca. 2–3 mm long, pubescent with short simple hairs, raphe ± glabrous.

##### Etymology.

The species epithet honours H. David Clarke who, while employed by the Biological Diversity of the Guiana Shield Program, National Museum of Natural History, Smithsonian Institution, was an adventurous and discerning plant collector in Guyana (see Kelloff et al. (2011)).

##### Distribution.

At present, known only from the Wassarai [Wassari] Mountains in southernmost Guyana near the Brazilian border (Kelloff et al. (2011): map 9, trip 18) where it was found in dense forest on red sandy clay.

##### Discussion.

This is the first record of *Callianthe* occurring in the Guianas. For convenience, *C.clarkei* was compared to *C.sylvatica*, but I am reluctant to consider the two species closely related. Their inflorescences and flower petals are quite different. In fact, the inflorescence of *C.clarkei* resembles that of several collections from Putumayo, Colombia that are associated here with *C.sylvatica* with doubt (see below).

#### ﻿2. Callianthe insignis

Taxon classificationPlantaeMalvalesMalvaceae

(Planch.) Dorr comb. nov.

63FE6621-04DF-5A39-A3C8-E8EEE685D31D

urn:lsid:ipni.org:names:77366029-1

Fig. 2

Abutilon insigne Planch., in Linden, Établ. Linden, Prix-courant 5: 4, 19. [Mar] 1850; Planchon, Fl. Serres Jard. Eur. 6: 41, t. 551. [6 Jun] 1850; Hooker, Curtis’s Bot. Mag. 81 [= ser. 3, 11]: t. 4840. 1855; Triana & Planchon, Ann. Sci. Nat., Bot., sér. 4, 17: 184. 1862; Linden & Planchon, Pl. Columb. 1: 46. 1874–75 [“1863”], as to name only. Type: Luxembourg [cultivated]. “Hort. Linden ex andibus Nov. Granat.,” s.d. (fl), *N. Funck & L.J. Schlim s.n.* (lectotype, here designated: MPU [MPU016978 as image!]).
Abutilon igneum hort., Garden (London, 1871–1927) 14: 474, fig. 1878, sphalm pro “*insigne*.”
Abutilon insigne ‘Duc de Malakoff’ De Bosschere, Rev. Hort. Belg. Étrangère 17: 127. 1891 (“var. Duc de Malakoff”). Type: Not designated.
Sida insignis Planch. ex Bellair & St.-Lég., Pl. Serre 80. 1900, nom. nud., pro syn.

##### Type.

Based on *Abutiloninsigne* Planch.

**Figure 2.**
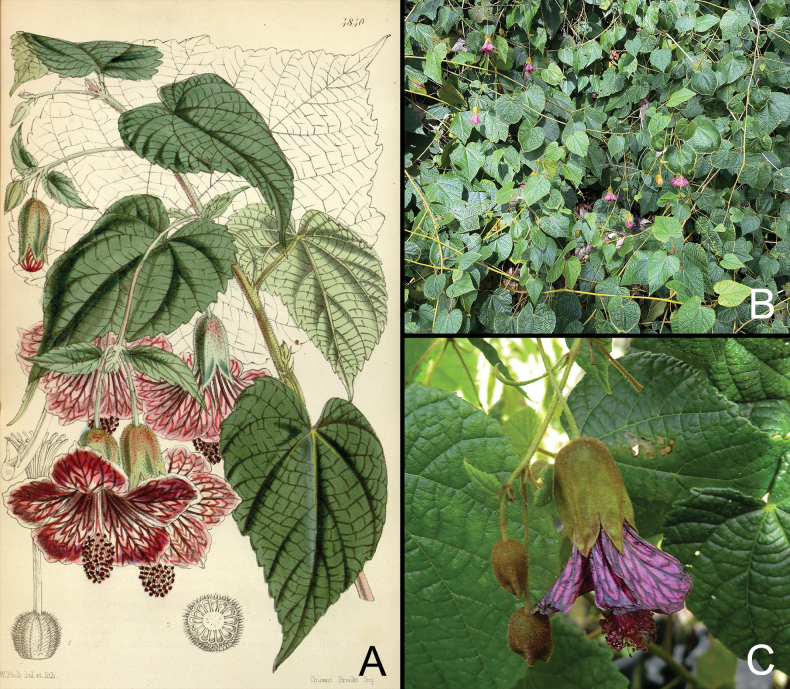
*Calliantheinsignis* (Planch.) Dorr. **A.** Illustration of *Abutiloninsigne* published in Curtis’s Botanical Magazine; **B.** Habit; **C.** Flower (Sources: **A.**Hooker (1855: t. 4840); **B.** Photograph by Glyn Church; **C.** Photograph courtesy of Woodleigh Nursery).

##### Distribution.

Found in the Andes of Colombia and/or Venezuela; further details wanting.

##### Additional material examined.

**Colombia** • Habitat in Andibus Columbiens, 1878 (fl), *J.H. Wibbe s.n.* [leg.?] (BR [BR0000031103756 as image!]).

##### Discussion.

When Planchon first validly published this name in a nursery catalogue distributed by Linden (1850: 4, 19), he announced that it would be illustrated that spring in the horticultural journal “Flore des serres et jardins d’Europe”. The index to Linden’s nursery catalogue gave an even more precise date for the promised illustration (“voir *Flore des serres*, février 1850”). There is, however, no illustration of *A.insigne* in the February issue of that journal and, for whatever reasons, one did not appear until several months later in the June issue (Planchon 1850: t. 551). Thus, while the illustration that Planchon promised may have existed when the name *A.insigne* first was published, it is not certain that it did exist and the plate’s status as original material is equivocal. Accordingly, the lectotype designated by Fryxell (2002: 95) is superseded here with a specimen from the Linden establishment.

Linden and Planchon (1874–1875) later cited a collection (“Linden-Funck et Schlim, n° 750”) of *Abutiloninsigne* from “Venezuela – Agua Obispo (prov. de Truxillo)”, but no specimens of this collection have been located and the description of 3-lobed leaves and the locality suggest the collection might, in fact, be the species described below as *Calliantheroseangelae* Dorr. In any case, apart from this collection and the Wibbe one cited above, no other wild-collected material of *A.insigne* has been reported or identified. It is unfortunate that the Rev. John Hermann Wibbe (1849–1878), who assembled a personal herbarium that included his own collections along with those gathered by others worldwide, elected to suppress the name of whomever collected his specimen of *A.insigne* and concealed precisely where it was collected.

De Bosschere (1891: 127), in discussing cultivated material of *Abutilon* seen in Ghent and Lièges, Belgium, recognised *A.insigne* ‘Duc de Malakoff’, based on a rather trivial petal colour difference. De Bosschere wrote that his cultivar had pinkish-purple petals with purple-carmine veins, while *A.insigne* as described by Planchon (1850) and Morren (1855) had soft pink or white petals with purple and carmine veins. Unfortunately, the cultivar name adopted by De Bosschere (1891) had been applied by horticulturists since 1870, at least, to a mottled-leaf (virus-infected) variety of *A.striatum* Dicks. ex Lindl. (Hibberd 1870).

Fryxell in Bernal et al. (2016: 2535) did not cite wild-collected material of *Abutiloninsigne* from Colombia, but reported that the species was cultivated in the Andes of Caldas and Cundinamarca. The voucher Fryxell cited for Cundinamaraca (*Sänchez & Linares 1776*, COL [COL000139180 as image!]), however, is not *A.insigne* nor is what is presumed to be Fryxell’s voucher for Caldas (i.e. *de Fraume & Alvarez y Gallego 379*, COL [COL000139181 as image!]). Both specimens can be referred to a complex of *Callianthe* cultivars that often are named for convenience as “A.×hybridum”.

Kearney (1958) wrote that *Abutiloninsigne* occurred in Venezuela, but did not cite a voucher. Dorr in Hokche et al. (2008) reported that *A.insigne* occurred in the State of Trujillo and probably also Táchira, Venezuela. The Trujillo record was based, in part, on material described below as *Calliantheroseangelae*. The Táchira record was taken from Bono (1996), but Bono’s brief description (“Flores rojas, ....”) and stated provenance (“Originaria de Asia Menor”) suggest it is not this species.

*Calliantheinsignis* is a distinctive plant with a scandent habit, rugose to slightly bullate leaf blades that are entire or faintly 3-lobed and coriaceous, pendulous inflorescences with flowers borne on long, articulated peduncles, pale lavender or white petals with dark purple veins, petals spreading at anthesis and a slightly exserted androecium. Despite our lack of knowledge regarding wild populations, *C.insignis* has been in cultivation since it was first discovered ca. 175 years ago and is included in standard horticultural references as *Abutiloninsigne* (Bailey 1949; Bailey and Bailey 1976; Everett 1980; Huxley et al. 1992; Griffiths 1994). The species commonly known as “Climbing Chinese lantern” currently is available in the trade in New Zealand, at least. Both Church Gardens and Woodleigh Nursery in New Plymouth cultivate this species, which was acquired from Hollard Gardens in Tarankai (Glyn Church, pers. comm.) (Fig. 2). The last-named nursery no longer has records regarding how it acquired the species.

A specimen in the Meisner Herbarium (NY [02339732]!) indicates *Abutiloninsigne* was cultivated in Brazil as early as 1854, but more recent material from Brazil has not been seen. The source of the Meisner material is stated to have been New Granada, but whether the plant came to Brazil via Luxembourg (Linden) or directly from northern South America is unknown.

Reports of *Abutiloninsigne* being cultivated in Costa Rica are suspect (Standley 1937; Fryxell 2007). Vouchers were not cited and the determinations reported in these floras cannot be verified.

#### ﻿3. Callianthe petiolaris

Taxon classificationPlantaeMalvalesMalvaceae

(Kunth) Donnell, Syst. Bot. 37(3): 720. 2012.

1FD3B060-E9C9-59A1-BCFC-1BA369532D3C

Abutilon petiolare Kunth, in H.B.K., Nov. Gen. Sp. [qu. ed.] 5: 273. 1822 [“1821”]; Ibid., Nov. Gen. Sp. [fol. ed.] 5: 213. 1822 [“1821”]. Type: Colombia. Nova Granada, sine loc., s.d. (fr), *F.W.H.A. Humboldt & A.J.A. Bonpland s.n.* (lectotype, here designated: P [P00679725 as image!]; isolectotype: P [P002285589 (= F neg. no. 35460) as image!]).
Sida petiolaris (Kunth) DC., Prodr. 1: 470. 1824. Type: Based on Abutilonpetiolare Kunth.

##### Type.

Based on *Abutilonpetiolare* Kunth.

##### Distribution.

Endemic to Colombia where it is found in the Cordillera Oriental; 1100–1300 m elev. Reports for Bolívar and La Guajira Departments (Fryxell in Bernal et al. (2016: 1540)), probably are incorrect since material from those Departments deposited in the Herbario Nacional Colombiano (COL) is misidentified and should be referred to *Bakeridesiaintegerrima* (Hook. f.) D. M. Bates.

##### Additional material examined.

**Colombia** • **Cundinimarca**: Mpio. de La Mesa, “Camino del Palmar,” al SE de La Mesa, 1100–1300 m elev., 16 May 1952 (fl, fr), *A. Fernández & L.E. Mora 1382* (COL [COL000139199 as image]). • Prov. de Tequendama, cerca de Tena, 1300 m elev., May 1853 (fl, fr), *J.J. Triana 3160* [*5292*] (BM [BM013837148 as image]). • Prov. de Bogotá, près de Tena, 1300 m elev., May 1853 (fr), *J.J. Triana 5292/3* (COL [COL000139196 as image]). • Prov. de Bogotá, près de Tena, 1300 m elev., 1851–1857 (fl, fr), *J.J. Triana s.n.* (K [K006358367 as image], NY, P [P06594555 as image]). • Tena, Andes de Bogotá, 1300 m elev., s.d. (fl, fr), *J.-E. Planchon s.n.* (MPU [MPU771539 as image]). • **Meta**: N^elle^. Grenade, Cordillera Orientale, La Meta, 1844 (fl, fr), *J. Goudot s.n.* (P [P06594554 as image]). • **Department unknown**: Sine loc., 1783–1808 (fl), *J.C. Mutis 958* (MA [MA667364 as image], MA [MA667365 as image], MA [MA667366 as image], US [01223748]).

##### Discussion.

The specimen selected as lectotype of the name *Abutilonpetiolare* was labelled by Kunth as “*Abutilonpetiolare* mihi” and is part of the “Herbier Humboldt & Bonpland.” It also has flower, fruit and mature mericarps. The isolectotype specimen, part of Bonpland’s “Herbier de l’Amérique équatoriale,” was labelled “*Sida* [illegible]” and has a reference in an unknown hand to the publication (quarto edition) of *A.petiolare*. It has a fruit and a flower.

This species lacks the horticultural appeal of other *Callianthe* species, yet it is distinctive. Pressed and dried material has a cinereous appearance; simple hairs on stems, petioles and pedicels are exceptionally long and visible without magnification; the leaf blade margin is coarsely serrate; leaf blade apices are long acuminate; petioles often clearly exceed leaf blades in length; and the petals are yellow.

#### ﻿4. Callianthe roseangelae

Taxon classificationPlantaeMalvalesMalvaceae

Dorr sp. nov.

E1EF3D4B-E840-5D2F-96EE-E6C377FF1D53

urn:lsid:ipni.org:names:77366032-1

Fig. 3

##### Diagnosis.

*Calliantheroseangelae* Dorr differs from *C.insignis* (Planch.) Dorr in leaf shape (non-apical leaf blades conspicuously 3-lobed versus unlobed or inconspicuously lobed), leaf surface (smooth versus rugose to slightly bullate), calyx shape (broadly campanulate versus tubular), calyx lobe shape (broadly versus narrowly triangular) and staminal column indumentum (stellate pubescent versus glabrous).

##### Type.

Venezuela • Trujillo: Municipio [“Distrito”] Boconó, abajo del Páramo de La Cristalina: Quebrada de La Cañada, 2400 m elev., 17 Feb 1973 (fl, fr), *J. Cuatrecasas, L. Ruiz-Terán, & M. López-Figueiras 28563* (holotype: US [04135468]!; isotypes: NY [04290815]!, US [04135467]!, US [04135469]!).

##### Description.

Scandent or vining shrubs with hanging branches; stems stellate-pubescent, glabrate in age. Leaves simple, entire; leaf blades differing in shape and size depending upon location on branchlets; apical leaves relatively smaller, ovate, 2–9 × 1.3–4.8 cm, unlobed or with a slight suggestion of 3 lobes, apices long acuminate, margin crenulate, bases cordate; non-apical leaves relatively larger, broadly ovate, 7–15 × 5–11 cm, 3-lobed with the lateral lobes diverging and ending in long acuminate apices, central lobes also long acuminate, margin crenulate, bases deeply cordate with sinuses to 2 cm deep; all leaves 7-nerved at the base, densely stellate-pubescent below with multi-rayed sessile hairs, rays ascending, ± colourless, leaf surface visible between hairs, stellate hairs on principal veins slightly larger, darker and more dense; scattered stellate-pubescent above with multi-rayed hairs, rays ± appressed or slightly ascending, leaf surface visible between hairs, rough to the touch, stellate hairs on principal veins slightly larger, darker and more dense; dark greyish-green above (fide *Cuatrecasas et al. 28563*), dull green below, smooth (not rugose nor slightly bullate), firmly membranous; petioles 1–6 cm long, densely stellate-pubescent; stipules ca. 5 × 9–10 mm, slightly falcate, stellate-pubescent, caducous. Flowers pendent, solitary in leaf axils, borne on slender peduncles 8–13 cm long (expanding to 22 cm long in fruit), stellate-pubescent, articulated 1–4 cm below the calyx, much more densely ferruginous stellate pubescent above the articulation; flower buds densely ferruginous stellate-pubescent, somewhat globose but coming to an apical point, sepals valvate. Involucel absent. Calyx broadly campanulate, 2–2.5 × 2–3.5 cm at anthesis, gamosepalous, 5-lobed, lobes broadly triangular, unequal in size and shape, ca. 0.5–1 cm × 0.7–1 cm, external surface densely stellate-pubescent with greenish-brown, brown or ferruginous hairs, internal surface light green, villous, with long simple hairs, nectariferous at base. Petals broadly spatulate, 4.5–5.5 × 2–3 cm, narrowing to a long claw, claw stellate-pubescent internally especially towards base, external surface of petals with scattered simple multicellular hairs, pink or whitish with strong purplish-red or deep rose branching veins, petals turning dark red when dry, evidently spreading slightly at anthesis. Staminal column ca. 4.3 cm long, lower 2 cm stellate-pubescent, filaments clustered apically, ca. 5 mm long, anthers greenish-brownish, not or very slightly exserted beyond the corolla. Styles ± equal to anther mass in length. Stigmas capitate. Fruit schizocarpic, ca. 2.5 × 4 cm, depressed-globose, calyx accrescent; mericarps ca. 8, rhomboid, ca. 2 × 1 cm, dorsally stellate-pubescent, inner wall glabrous, dehiscent. Seeds semi-deltoid, ca. 3 × 2 mm, pubescent, with simple trichomes, trichomes denser near hilum.

##### Etymology.

The species epithet honours Rose Angela Gulledge who has made substantial organisational contributions to the Flora of Guaramacal (Venezuela) project (Dorr et al. 2001; Dorr 2014; Dorr and Niño 2024), which treats the vascular plants of a national park close to the type locality of this new species.

##### Distribution.

Endemic to Venezuela where it has been found only in cloud forest in the Venezuelan Andes near the Páramo La Cristalina northwest of Boconó; 2300–2500 m elev.

##### Additional material examined.

**Venezuela** • **Trujillo**: Carretera vieja entre Trujillo y Boconó, entre Urbina y San Rafael, 32 km from Trujillo, 2300–2500 m elev., 3–4 Sep 1966 (fl), *J.A. Steyermark & M. Rabe 97235* (NY [04290815], US [01217620]).

##### Discussion.

**Figure 3.**
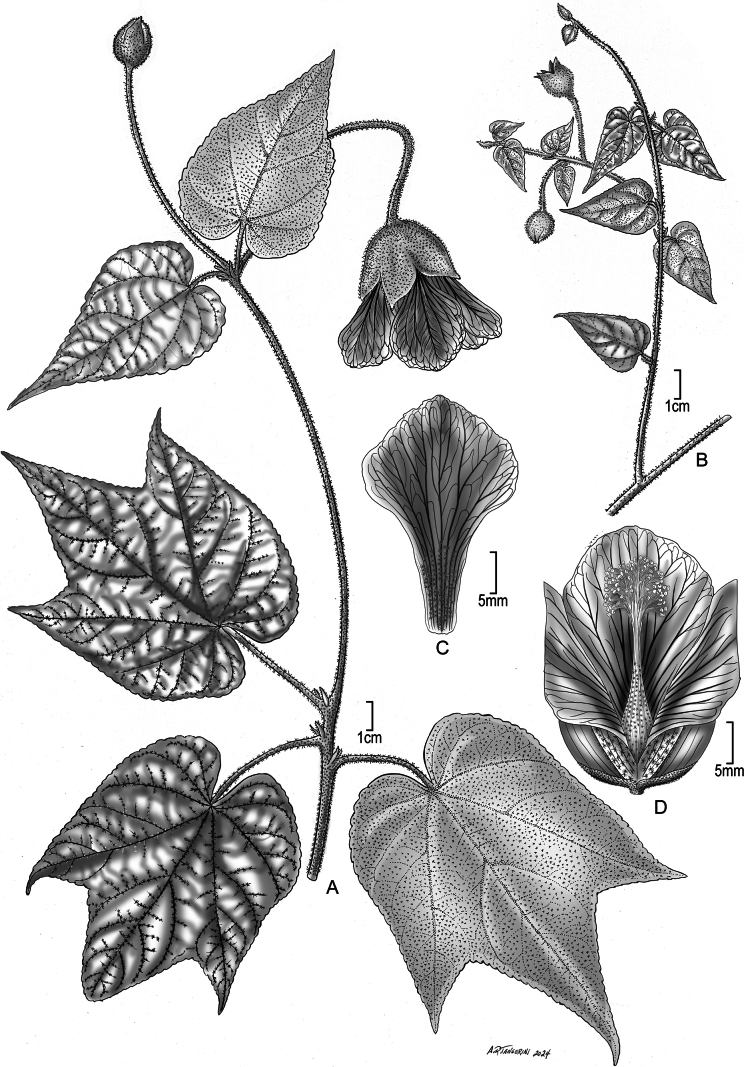
*Calliantheroseangelae* Dorr. **A.** Habit, branch with entire and 3-lobed leaves; **B.** Habit, apical portion of branch with reduced entire leaves; **C.** Petal; **D.** Flower, petals removed to show anther column and anthers (Vouchers: **A.***Cuatrecasas et al. 28563*, US [04125467]; **B.***Steyermark & Rabe 97235*, NY [04290815]; **C, D.***Cuatrecasas et al. 28563*, US [04125468].

The only fruit available for inspection had already shed seed and the number of seeds per mericarp could not be established apart from there clearly being more than one. The inner walls of the dehisced mericarps are glabrous, which contradicts one of the morphological characters that Donnell et al. (2012) used to define the genus *Callianthe*. Likewise, the staminal column in *C.roseangelae* is stellate pubescent below (Fig. 3D), which contradicts another morphological character that Donnell et al. (2012) used to define the genus *Callianthe*. However, the flowers agree with *Callianthe* as the petal veins are impressed and a darker colour than the petals. Additionally, the petals are not rotate like those of *Abutilon* s.str. and instead, while the petal apices are spreading at anthesis, their bases remain strongly imbricate.

The paratype collection originally was identified (“ex char.”) as *Abutiloninsigne* (≡ *Calliantheinsignis*). While *C.roseangelae* and *C.insignis* are morphologically similar and possibly closely related, they are distinct as noted in the diagnosis. Presumably, these two species also are separated geographically, but because the precise range of the latter species is unknown, this remains conjecture. In any case, whether it is due to geographic isolation, infrequent flowering or some other factor, it is remarkable that two spectacularly beautiful Andean species in a group long favoured by horticulturists are so infrequently collected.

#### ﻿5. Callianthe sylvatica

Taxon classificationPlantaeMalvalesMalvaceae

(Cav.) Dorr, in Jørgensen et al., Monogr. Syst. Bot. Missouri Bot. Gard. 127: 1272. 2014.

7FDB0EEB-7881-5146-8E41-0F2172DBA07A

Sida sylvatica Cav., Diss. 2: 56. 1786 (“Sylvatica”); Diss. 5: 276, t. 133, fig. 2. 1788. Type: Peru. “in sylvis prope flumen Maragnon,” s.d. (fl), *J. Dombey s.n.* (holotype: MA [MA-656281 as image!]).
Abutilon geminiflorum Kunth, in H.B.K., Nov. Gen. Sp. Pl. [qu. ed.] 5: 274, t. 474. 1822 [“1821”]; Ibid. [fol. ed.] 5: 213, t. 474. 1822 [“1821”]. Type: Venezuela. Caracas, [Jan-May/Jun 1800] (fl, fr), *F.W.H.A. Humboldt & A.J.A. Bonpland 1132* [“Bonpl. mss. n. 1132”] (lectotype, here designated: P [P00679726 as image!]; isotype: B [B-W 12680-01 0 (= F neg. no. 9795) as image!]).
Sida geminiflora (Kunth) DC., Prodr. 1: 470. 1824. Type: Based on Abutilongeminiflorum Kunth.
Abutilon dianthum C. Presl, Reliq. Haenk. 2(2): 114. 1835. Type: Peru. in montibus huanoccensibus Peruviae, s.d. (fl), *T. Haenke s.n.* (second step lectotype, here designated: PR [sheet no. 612209 as image!]; isolectotypes: HAL [HAL0118315 as image!], M [M-0211073 as image!], PR [sheet no. 612210 as image!], W [sheet no. W 0032650 (= F neg. no. 32630) as image!]).
Abutilon sylvaticum (Cav.) C. Presl, Reliq. Haenk. 2(2): 114. 1835 (“*sylvatico*”). Type: Based on Sidasylvatica Cav.
Sida diantha (C. Presl) D. Dietr., Syn. Pl. 4: 856. 1847. Type: Based on Abutilondianthum C. Presl.
Abutilon sylvaticum (Cav.) K. Schum., in Martius, Fl. Bras. 12(3): 418. 1891 (“*silvaticum*”), isonym. Type: Based on Sidasylvatica Cav.
Abutilon laxum Rusby, Mem. New York Bot. Gard. 7: 296. 1927. Type: Bolivia. Rio Bopi Valley, 3000 ft elev., 11 Sep 1921 (fl, fr), *H.H. Rusby 658* (holotype: NY [00188230]!; isotypes: BKL [00000980 as image!], K [K000328757 as image!]).
Abutilon woronowii Ulbr., Notizbl. Bot. Gart. Berlin-Dahlem 11(107): 520. 1932 (“*Woronowii*”); Ulbrich, Notizbl. Bot. Gart. Berlin-Dahlem 14: 357. 1939. Type: Venezuela. Aragua: Colonia Tovar, 21 Oct 1926 (fl), *G.J.N. Woronow 7480* (lectotype, here designated: LE-n.v.).
Abutilon sylvaticum subsp. genuinum R.E. Fr., Kongl. Svenska. Vetenskapsakad. Handl., ser. 3, 24(2): 7. 1947, nom. inval.
Abutilon sylvaticum subsp. buchtienii R.E. Fr., Kongl. Svenska. Vetensk. Acad. Handl., ser. 3, 24(2): 8. 1947 (“Buchtienii”). Type: Bolivia. Cotaña am Illimani, 2450 m elev., Nov 1911 (fl, fr), *O. Buchtien 3206* (holotype: S [S 12-17687 as image!]; isotypes: NY [054955168]!, US [01049726]!, US [01105686]!).
Abutilon sylvaticum subsp. klugii R.E. Fr., Kongl. Svenska. Vetensk. Acad. Handl., ser. 3, 24(2): 8. 1947 (“*Klugii*”). Type: Peru. San Martín: Zepelacio, near Moyobamba, 1100 m elev., Jul 1934 (fl, fr), *G. Klug 3749* (holotype: S [S 12-17685 as image!]; isotypes: CAS [0000019 as image!], F [F0042086F as image (= F neg. no. 56220)!], MO-n.v., NY [04955169]!, US [00098289]!).
Callianthe geminiflora (Kunth) Donnell, Syst. Bot. 37(3): 719. 2012. Type: Based on Abutilongeminiflorum Kunth.
Callianthe laxa (Rusby) Donnell, Syst. Bot. 37(3): 719. 2012. Type: Based on Abutilonlaxum Rusby.
Gaya sylvatica (Cav.) Krapov., Bonplandia (Corrientes) 21(1): 72. 2012. Type: Based on Sidasylvatica Cav.
Abutilon prolificum Rusby, nom. nud., in sched.

##### Type.

Based on *Sidasylvatica* Cav.

##### Distribution.

Found in the Cordillera Oriental of Colombia and the Cordillera de la Costa and Andes of Venezuela; 700–2300 m elev. This species also occurs in the Andes of Ecuador, Peru and Bolivia. Guézou et al. (2007) reported that *Callianthesylvatica* (as *Abutilondianthum*) was adventive in the Galapagos Islands and recommended that it be eradicated there.

##### Additional material examined.

**Colombia** • **Boyacá**: Mpio. Santa Maria, Sendero Ecologica: Aiku-Yala, 700–800 m elev., 21 Aug 2008 (fl, fr), *J.L. Fernández-Alonso 26978* (COL [COL000373753 as image]). • Moniquirá, Belchite, 05°55'N, 073°35'W, 1700–2000 m elev., 30 Dec 1995 (fl, fr), *F. González 3528* (NY [1579546]). • Municipio de Santa María, vereda Chalicana, Sendero Ecológico Hyka-Quye, 04°53.5'N, 073°15.9'W, 900 m elev., 1–10 Sep 2010 (fl, fr), *M.F. González et al. 520* (COL [COL000380272 as image], COL [COL000380512 as image], NY [03066136 as image]). • **Cundinimarca**: bei Anolaima, 2000 m elev., Jan 1883 (fl), *F.C. Lehmann 2506* (BM [BM013837154 as image], US [01223674]). • Chiefly near Bogota, ca. 1850 m elev., Aug 1915 (fl, fr), *Mrs. J.* [sic] *A. Tracey 63* (K [K006358152]). • Cachipay, 5000–6000 ft elev., 1922 (fl), *Mrs.* [*I.A.*] *Tracey 480* (K [K006358095 as image]). • **Santander**: Vicinity of La Baja, 2200–2600 m elev., 14–28 Jan 1927 (st, immature), *E.P. Killip & A.C. Smith 17120* (NY). • Vicinity of La Baja, 2200–2600 m elev., 14–28 Jan 1927 (fl), *E.P. Killip & A.C. Smith 17132* (NY [04290816], US [01223672]). • Between El Roble and Tona, 1500–1900 m elev., 17 Feb 1927 (im fr), *E.P. Killip & A.C. Smith 19424* (GH [00255417 as image], NY, US [01223672]). **Venezuela** • **Aragua**: Colonia Tovar, 1800–2000 m elev., Dec 1924 (fl, fr), *A. Allart 316* (NY, US [01223691]). • Muy cerca de la Colonia Tovar, entrando por La Lagunita, ca. 1700 m elev., 21 Oct 1972 (fl), *C.E. Benítez de Rojas 1544* (F [V0224938F as image]). • Prope coloniam Tovar, 1854–5 (fl), *A. Fendler 98* (BR [BR0000013462093 as image], K [K 006358109 as image], NY [05125048], P [P06595596 as image]). • Distrito Ricaurte, Fila El Socorro-Topo El Paují, al SE de La Victoria, 1300–1500 m elev., 25 Nov 1999 (fl, fr), *W. Meier et al. 5892* (US [01223693]). • Colon. Tovar, Columbia [sic], Sep 1847 (fl), *M. Moritz 815* (K [K006358108 as image]), Colonia Tovar, 1865 (fl), *M. Moritz 815* (BM [BM013837155 as image]). • Colonia Tovar, 1852 (fl), *M. Moritz s.n.* (P [P06595595 as image]). • Colonia Tovar, 5 Jan 1941 (fl), *F. Tamayo 1572* (US [01223694]). • Cerca de La Cumbre de Choroní, 1400 m elev., s.d. (fl), *Ll. Williams 10490* (F [V0224937F as image], US [01223695]). • **Barinas**: 12 km N of Baramitas [sic, Baranitas], 700 m elev., *R. Jones 9* (NY). • **Distrito Capital**: Serranias del Avila, 28 Dec 1943 (fl), *T. Lasser 980* (US [01223696]). Cerro El Avila, Cabecera del Río Mata de Plátano, 2000 m elev., 1 Oct 1976 (fl), *B. Manara s.n.* (NY [04290818]). • Parque Nacional El Avila, Cerro El Avila, vertiente sur, 10 Nov 1982 (fl), *W. Meier 3035* (US [01183942]). • Parque Nacional El Avila, 1500–1700 m elev., 26 Nov 2000 (fl), *W. Meier et al. 7522* (US [01223692]). • Chacaito Gorge, around Caracas, 800–1000 m elev., 11 Dec 1921 (fl), *H. Pittier 9931* (NY, US [01223698], US [01223699]). • Parque Nacional El Avila, entre la toma de agua de La Zamurera y Papelón, 29 Nov 1986 (fl), *C.E. de Rojas & F. Rojas 3557* (NY). • W and SW-facing slopes of Cordillera del Avila, above Caracas, between Los Venados and Guayabo Mocho, 1675–2075 m elev., 28 Dec 1943 (fl, fr), *J.A. Steyermark 55054* (F [V0224936F as image], NY [04290817], US [01223700]). • Los Venados, ca. 1500 m elev., 1 Oct 1940 (fl), *C. Vogl 478* (F [V0224935F as image]). • **Mérida**: Carretera Mucubají - Barinas, más abajo de Sto. Domingo, 10 Nov 1952 (fl, fr), *L. Aristeguieta 1049* (NY [05125047], US [01223701]). • Distr. Rangel, Barinas-Mérida highway, 6.7 km E of La Mitisus (08°53'N, 070°39'W) (5.2 km W of Mérida/Portuguesa state line), ca. 1600 m elev., 7 Nov 1990 (fl), *L.J. Dorr & L.C. Barnett 7650* (K [K006358134], NY [05125046], PORT). • 5 km W of Mérida-Barinas boundary on road to Barinas, 1680 m elev., 9 Jan 1989 (fl), *W. Hahn & F. Grifo 5073* (K [K006358124 as image], NY, U [U.1363669 as image], US [00511982]). • **Miranda**: Los Teques, in German plantations (Parque de los Bárbaros), 1400–1500 m elev., Dec 1917 (fr), *H. Pittier 7604* (US [01223697]). • **Trujillo**: Distrito Bocono, La Morita, above the Río Sagurás, S of Campo Elías and ca. 3 km W of Trujillo-Portuguesa state line, 2300 m elev., 4 Jun 1998 (fr), *L.J. Dorr et al. 5374* (NY [04290819], PORT). • Distrito Carache, Alrededores de Cajingo (00°37'N, 070°09'W), Quebrada Cajingo, 2000 m elev., 1 Nov 1987 (fl), *R. Rivero & W. Diaz 1391* (FLAS [sheet no. 169022 as image], NY [00393841]).

The following collections also may belong here. They differ principally in their inflorescences where flowers are found not only in the distal-most axils of leaves, but also in the 4 or 5 uppermost leaf axils below the terminal one.

##### Material examined.

**Colombia** • **Putumayo**: Municipio de Mocoa, Vereda Bajo Afán, Serranía El Churumbelo, sector Nor Oriental, orilla (margen izquierda) del río Mocoa, 1°11'37"N, 76°38'49"W, 630 m elev., 4 Oct 2000 (fr), *D. Cárdenas et al. 12155* (COAH [sheet no. 35566 as image]). • San José, Río Putumayo, 5 Aug 1899 (fl), *T.A. Sprague 614* (BM [BM013837151 as image], K [K006358094 as image]).

##### Discussion.

Presl (1835) transferred *Sidasylvatica* Cav. to *Abutilon*, but Presl’s action was overlooked by Schumann (1891) who nonetheless saw the same relationship. Schumann’s interpretation of the proper placement of this Peruvian species was followed by Garcke (1893), Macbride (1956) and Kearney (1958), the first reviewing Schumann’s treatment and the last two revising the Peruvian and South American species of *Abutilon*, respectively. Fryxell (1992), who treated the Ecuadorean species of *Abutilon*, however, adopted *A.dianthum* C. Presl as the name of this species since he argued that the name *S.sylvatica* could not be applied to a species of *Abutilon* because Cavanilles described the fruit as having uniovulate carpels. Fryxell (1992) circumscribed *Abutilon* as having (2–)3–6 ovules per carpel and, while his description of *A.dianthum* omitted a statement regarding ovule number, it has 7–9 ovules per carpel, which aligns it with the pluriovulate species of *Abutilon* that Donnell et al. (2012) transferred to *Callianthe*. Fuertes and Fryxell (1993) and Fryxell (2002) repeated Fryxell’s (1992) argument regarding *S.sylvatica* and even stated that the identity and generic placement of Cavanilles’s plant were problematic. Krapovickas (2012), relying on the original description of Cavanilles (1786) and a plate that Cavanilles (1788) published subsequently, also focused on the statement (and illustration) by Cavanilles that the carpels were uniovolute and opined on the basis of this character that the only possible generic placement was *Gaya* Kunth.

These arguments concerning the identity of *Sidasylvatica* are curious because the holotype of *S.sylvatica*, a specimen collected by Joseph Dombey (MA [MA-656281]), which also appears to subsequently have served as the basis of the plate that Cavanilles (1788: t. 133, fig. 2) published to illustrate *S.sylvatica*, exists. The rules of the International Code of Nomenclature (Turland et al. 2025: Arts. 9.1, 9.14) are clear that the type, not a description, fixes the application of the name concerned. Thus, in this case, while the description of the carpels by Cavanilles (1786) is at variance with the definition of *Abutilon* (and *Callianthe*), the holotype of *S.sylvatica* nonetheless represents a species best placed in *Callianthe* because material from Peru that matches the type is pluriovulate with more than six ovules per carpel. Interestingly, the holotype of *S.sylvatica* does not now have fruit, which begs the question as to the source of this discordant element in Cavanilles’s (1786, 1788) description and illustration.

Fuertes and Fryxell (1993: 663) stated that “an apparent isotype” of *Sidasylvatica* was found in the general herbarium in Madrid (MA [MA-265804 as image!] [= F neg. no. 29760]), but the locality on the label of this specimen, Cochexo (or Tochexo), differs from that of the holotype and there is no evidence that the specimen was collected by Dombey. It clearly represents the same taxon, but cannot be considered type material. Krapovickas (2012) confused this specimen with the holotype of *S.sylvatica*, citing the correct locality and collector, but “MA 29760 [sic], foto F 29760” as the holotype.

Fryxell (1992: 10) designated a Haenke collection in Prague (PR) as the lectotype of the name *Abutilondianthum*, but he failed to distinguish between two duplicates of that collection held in PR (Turland et al. 2025: Art. 9.17). Consequently, a second-step lectotype is necessary and is designated here. The locality on the lectotype label matches exactly that given in the prootologue. The locality on the isolectotype labels is slightly different (viz. “Peruviae”), but the handwriting is identical on four of the five specimens cited. The label on the specimen in Munich (M) was copied by a different person.

Ulbrich (1932) indicated that the type of *Abutilonworonowii* was a collection made in Venezuela, “Colonia Tovar (bluhend 21. Oktober 1926 – G. Woronow n. 7480 typ.)”, which was deposited in Berlin (B). This specimen subsequently was destroyed in World War II and consequently a duplicate of the type, which Ulbrich (1939) noted later and indicated was deposited in St. Petersburg (LE), is here designated as lectotype of this name. Although the type of *A.woronowii* was not examined in the present study, two paratypes (*Allart 316* and *Pittier 9931*) (Ulbrich 1932, 1939) were studied and they are indistinguishable from *C.sylvatica*.

‘*Abutilonprolificum*’ is a herbarium name that Rusby wrote on sheets of one of his Bolivian collections, *Rusby 737* (GH [0052675 as image], K [K000328758 as image], US [01217797]), which was made during the Mulford Biological Exploration of the Amazon Basin.

*Callianthesylvatica* is interpreted here as an erect shrub with ovate to broadly ovate leaves, oblique to cordate bases, acute to acuminate apices, crenate-dentate margin and stellate-pubescent (often ferruginous) indumentum above and below; flowers solitary (or paired) in uppermost leaf axils, drooping or pendent; peduncles exceeding subtending petioles in length; calyx ca. half-lobed, ferruginous stellate-pubescent; petals spatulate, clawed, pale to bright yellow (or white), with impressed veins of the same colour; staminal column glabrous; schizocarps with ca. 10–12(–16) mericarps, persistent calyx; and pubescent seed.

Although a number of authors (e.g. Candolle (1824); Don (1831); Schumann (1891); Baker (1893); Fryxell (2002)) have recognised both *Callianthesylvatica* and *C.geminiflora* (Kunth) Donnell or their respective synonyms, there is no clear distinction between the former species described from Peru and the latter described from Venezuela. At best, the Venezuelan species can be described as having slightly more oblique leaves than those of *C.sylvatica*. Similarly, while Kearney (1958) suggested *Abutilonlaxum* Rusby was a synonym of *A.sylvaticum* (= *C.sylvatica*), Donnell et al. (2012) recognised it as *C.laxa* (Rusby) Donnell. There is nothing to distinguish *C.laxa* from *C.sylvatica* and Dorr in Jørgensen et al. (2014: 791), who reviewed the Bolivian taxa of Malvaceae, listed the former as a synonym of the latter.

Donnell et al. (2012) noted that species relationships within their *Callianthe* and *Bakeridesia* s.str. clades are poorly resolved, but did not elaborate on the possible reasons.

###### ﻿Excluded name

#### ﻿ Callianthe purpurascens

Taxon classificationPlantaeMalvalesMalvaceae

(Link) Dorr comb. nov.

8EE4955D-4CBA-5C4F-B7A4-0F5AD622BA97

urn:lsid:ipni.org:names:77366035-1

Sida purpurascens Link, Enum. Pl. Hort. Berol. Alt. 2: 206. 1822. Type: Rio de Janeiro: Commun près Rio de Janeiro, s.d. (fr), *A. St.-Hilaire 73B* (neotype, here designated): US [00098237]!; isoneotypes: B† [= F neg. no. 9266], MPU [MPU013687 as image!], P [P02285558 as image! (= F neg. no. 35451 p.p.)], P [P02285559 as image!], P [P02285560 as image (= F neg. no. 35451 p.p.)!]; probable isoneotype: F [F0062850F as image!]).
Sida rosea Link & Otto, Icon. Pl. Select.: 71, t. 32. 1825; Hooker, Curtis’s Bot. Mag. 69 [= ser. 2, 6]: t. 3150. 1832. Type: The plate (t. 32) published with the original description (lectotype, here designated).
Sida speciosa Willd. ex Spreng., Syst. Veg., ed. 16, 3: 119. 1826, nom. illeg. superfl.
Abutilon esculentum A. St.-Hil., A. Juss. & Cambess., Pl. Usuel. Bras.: t. 51. [16 Jun] 1827 [“1824”]; A. St.-Hil., Fl. Bras. Merid. [qu] 1(6): 204. [18 Jul] 1827 [“1825”]; Ibid., Fl. Bras. Merid. [fol] 1: 160. 1827 [“1825”]. Type: Brazil. Rio de Janeiro: Commun près Rio de Janeiro, s.d. (fr), *A. St.-Hilaire 73B* (lectotype, designated by Fryxell (2002: 90): P [P02285558 as image! (= F neg. no. 35451 p.p.)]; isolectotypes: B† (= F neg. no. 9266), MPU [MPU013687 as image!], P [P02285559 as image!], P [P02285560 as image! (= F neg. no. 35451 p.p.)], US [00098237]!); probable isolectotype: F [F0062850F as image!]).
Abutilon carneum A. St.-Hil., Fl. Bras. Merid. [qu] 1(6): 205. [18 Jul] 1827 [“1825”]; Fl. Bras. Merid. [fol] 1: 160. 1827 [“1825”]. Type: Brazil. Rio de Janeiro: Bord du Riv. das Ostras [sic], s.d. (fl, fr), *A. St.-Hilaire 163* (second step lectotype, here designated: P [P02285549 as image! (= F neg. no. 35448 p.p.)]; isolectotypes: MPU [MPU017039 as image!], P [P02285551 as image! (= F neg. no. 35448 p.p.)]); probable isolectotypes: F [F0062849F as image!], P [P02285550 as image!]).
Abutilon speciosum (Willd. ex Spreng.) G. Don, Gen. Hist. 1: 502. 1831, comb. illeg.
Rosa speciosa Hook., Curtis’s Bot. Mag. 69 [= ser. 2, 6]: t. 3150. 1832, sphalm. pro Sidaspeciosa Willd. ex Spreng.
Sida esculenta (A. St.-Hil., A. Juss. & Cambess.) Steud., Nomencl. Bot., ed. 2, 2: 577. 1841. Type: Based on Abutilonesculentum A. St.-Hil., A. Juss. & Cambess.
Sida hilaireana Steud., Nomencl. Bot., ed. 2, 2: 577. 1841 (“Hilaireana”), nom. nov. Type: Based on Abutiloncarneum A. St.-Hil.
Abutilon virens A. St.-Hil. & Naudin, Ann. Sci. Nat., Bot., sér. 2, 18: 48. 1842. Type: Brazil. Minas Gerais [“Minas Geraës”]: Sine loc., 1838 (fr), *P. Claussen 122* (lectotype, here designated: P [P02285617 as image!]; isolectotypes: G-DEL, P [P02285616 as image!]).
Sida carnea (A. St.-Hil.) D. Dietr., Syn. Plant. 4: 853. 1846, comb. illeg.
Abutilon purpurascens (Link) K. Schum., in Martius, Fl. Bras. 12(3): 419. 1891. Type: Based on Sidapurpurascens Link.
Bakeridesia purpurascens (Link) Monteiro, Anais Soc. Bot. Brasil 5: 436. 1956. Type: Based on Sidapurpurascens Link.
Bakeridesia esculenta (A. St.-Hil., A. Juss. & Cambess.) Monteiro, Anais Soc. Bot. Brasil 23: 127. 1973 [“1972”]. Type: Based on Abutilonesculentum A. St.-Hil., A. Juss. & Cambess.

##### Type.

Based on *Sidapurpurascens* Link.

## ﻿Discussion

Nominally, there is no reason to discuss a species endemic to Brazil in a paper focused on the northern South American species *Callianthe*. However, the extensive synonymy of *C.purpurascens* (Link) Dorr includes one name, *Sidaspeciosa* Willd. ex Spreng., associated with a Humboldt collection made in “Cumana”, which is the capital of Sucre State in northern Venezuela.

Original material of *Sidapurpurascens* Link does not appear to exist as noted by Schumann (1891), Garcke (1893) and Fryxell (2002). Consequently, a neotype is designated here and the selection is intended to fix the interpretation first proposed by Schumann (1891) that *S.purpurascens* and *Abutilonesculentum* A. St.-Hil. et al. are synonyms. The specimen selected as neotype agrees with the original description, which is that of a pubescent plant with crenate-denticulate leaves and clawed petals.

*Sidaspeciosa* is a superfluous name for *S.rosea* Link & Otto, which was described from Brazil. When Sprengel (1826) published the former name, he cited material from Brazil and Venezuela (“*Brasil. Cumana*. Humb.”). The Brazilian element clearly is *S.rosea*, which was cultivated in Berlin from seed sent from Brazil. The Venezuelan element (“*Cumana*. Humb.”), a Humboldt specimen, has not been located and there are otherwise no records of *S.rosea* from Venezuela. It may be that the Venezuelan element is the isotype of *Abutilongeminiflorum* (= *Callianthesylvatica*) in Berlin (B -W 12680 -01 0), which was collected by Humboldt & Bonpland in “Caracas” ca. 400 km to the west of Cumaná. Subsequent authors (Fryxell 2002; POWO 2025) failed to note that *S.speciosa* was a superfluous name and incorrectly considered it to be the “basionym” of *A.speciosum* (Willd. ex Spreng.) G. Don, comb. illeg. or treated it as a heterotypic synonym of *Bakeridesiaesculenta* (A. St.- Hil., A. Juss. & Cambess.) Monteiro. Donnell et al. (2012) suggested that the basionym of *B.esculenta*, *A.esculentum* A. St.- Hil., A. Juss. & Cambess., might be a *Callianthe*. Their suggestion has merit and is adopted here, but *Sidapurpurascens* is an older name and is the basionym of the new combination.

Although Fryxell (2002: 86) designated a specimen in Paris (P) as lectotype of *Abutiloncarneum* A. St.-Hil., he failed to distinguish between two of the three sheets then in that herbarium. He, however, indicated that the specimen designated here as lectotype was one of two “isotypes”. Kearney (1958) accepted *A.carneum* as a distinct species, but his opinion has not been followed.

Hortus Third (Bailey and Bailey 1976), ignoring the fact that *Abutilonspeciosum* (Willd. ex Spreng.) G. Don was based on an illegitimate superfluous name, speculated that “*A.speciosum* G. Don” was either “*A.hybridum* hort. ex Siebert & Voss” (= *Callianthe* cv.) or *A.pictum* (Gillies ex Hook. & Arn.) Walp. (≡ *Callianthepicta* (Gillies ex Hook. & Arn.) Donnell). Both suggestions clearly are incorrect.

The transfer of *Abutiloncarneum* A. St.-Hil. to *Sida* L. is blocked by the earlier *Sidacarnea* DC. and, thus, *S.hilaireana* Steud. is a replacement name. Likewise, *S.carnea* (A. St.-Hil.) D. Dietr. is an illegitimate combination because of the earlier *S.carnea* DC. Both the replacement name and the illegitimate combination were omitted by Fryxell (2002) from his *Abutilon* nomenclator.

When St.-Hilaire and Naudin (1842) published *Abutilonvirens*, they expressed doubt that it was distinct from *Sidarosea* writing “Abutilonvirens – *Sidarosea* Link et Otto, *Ic. select*. t. 32? – Hook. *Bot. Mag.* 3150?”. *Sidarosea*, in turn, cannot be distinguished from *S.purpurascens*.

Finally, although Schumann (1891) included *Sidatriflora* Vell. in his synonymy of *Abutilonpurpurascens*, he expressed doubt presumably because the Vellozo (1829) protologue mentions an entire leaf margin, while that of *A.purpurascens* (≡ *Callianthepurpurascens*) is crenate-denticulate. Yoshikawa and Pastore in Coutinho et al. (2025), however, considered Vellozo’s species and *C.latipetala* (G.L. Esteves & Krapov.) Donnell to be conspecific, ignoring the crenate-serrate leaf margin of the latter and focusing on what they argued was a distinctive shared feature, a change in petal colour once the flowers have fallen. This last character is somewhat tenuous as such petal colour changes in fallen flowers seems to be common amongst not only *Callianthe*, but also Malvaceae species.

## Supplementary Material

XML Treatment for Callianthe clarkei

XML Treatment for Callianthe insignis

XML Treatment for Callianthe petiolaris

XML Treatment for Callianthe roseangelae

XML Treatment for Callianthe sylvatica

XML Treatment for Callianthe purpurascens

## ﻿Additional information

### Use of AI

No use of AI was reported.

### Author ORCIDs

Laurence J. Dorr https://orcid.org/0000-0001-7157-363X

### Data availability

All of the data that support the findings of this study are available in the main text.
